# Ischemic Heart Disease in the Context of Different Comorbidities

**DOI:** 10.3390/life12101558

**Published:** 2022-10-07

**Authors:** Irina-Iuliana Costache, Bogdan-Mircea Mihai, Minerva Codruta Badescu

**Affiliations:** 1 Department of Internal Medicine, “Grigore T. Popa” University of Medicine and Pharmacy, 16 University Street, 700115 Iasi, Romania; 2 Cardiology Clinic, “St. Spiridon” County Emergency Clinical Hospital, 1 Independence Boulevard, 700111 Iasi, Romania; 3 Unit of Diabetes, Nutrition and Metabolic Diseases, “Grigore T. Popa” University of Medicine and Pharmacy, 16 University Street, 700115 Iasi, Romania; 4 Clinical Center of Diabetes, Nutrition and Metabolic Diseases, “St. Spiridon” County Emergency Clinical Hospital, 1 Independence Boulevard, 700111 Iasi, Romania; 5 III Internal Medicine Clinic, “St. Spiridon” County Emergency Clinical Hospital, 1 Independence Boulevard, 700111 Iasi, Romania

Ischemic heart disease (IHD) is a leading cause of morbidity and mortality worldwide. Since coronary atherosclerosis is its main substrate, IHD generally affects the geriatric population [1]. As aging is associated with an increased number of comorbidities, IHD further negatively influences these patients’ quality of life and longevity.

Ilie et al. debated the strong relationship between frailty and chronic coronary syndromes [2]. They discussed the mechanisms of the bidirectional interaction between frailty—a complex geriatric syndrome with many phenotypes—and increased risk of ischemic coronary events. They also highlighted the key roles of sarcopenia, systemic inflammation, endothelial dysfunction, and impaired lipid and glucose metabolisms. The authors also provided useful guidance for risk assessment, diagnosis, and treatment of old and frail patients with stable IHD.

Epidemiological studies have shown a decrease in the age at which risk factors for atherosclerosis begin to accumulate. In this context, the age at which the incidence and prevalence of IHD begin to increase has shifted to younger ages, causing the impact of the disease on public health to become even greater [3]. Approximately 1.72% of the world’s population is currently affected by IHD, and the prevalence is still rising. As the global distribution map of IHD demonstrates, Eastern European countries contribute by far the highest prevalence [4].

Although myocardial infarction is only the tip of the iceberg, its consequences have a significant negative impact on morbidity and mortality in patients with IHD. Leancă et al. [5] provided an excellent review on adverse cardiac remodeling in survivors of acute myocardial infarction. The authors performed an in-depth analysis of the pathophysiological mechanisms involved in this process, and systematized the complex methods of evaluation by biomarkers and both conventional and high-performance imaging. The main goals of treatment were to avoid the progression of IHD to heart failure and to reduce mortality. The article provides strong evidence to guide individualized treatment aimed at counteracting adverse ventricular remodeling and promoting reverse ventricular remodeling.

Cancer is highly prevalent in the elderly, and cardiac toxicity of antineoplastic agents is currently a topic of major interest. Badescu et al. have shown that some commonly used oncological treatments have adverse effects on coronary arteries, as they induce endothelial dysfunction, coronary artery spasm, thrombosis, and fibrosis [6]. These harmful structural and functional changes lead to cardiovascular events that require reduction or temporary or permanent stop of antineoplastic therapy, and all of these events have a negative impact on cancer outcomes. The authors provided a comprehensive, systematized, structured, and up-to-date analysis of the available literature regarding the optimal measures to mitigate the toxic effects of major antineoplastic therapies on coronary arteries. Sorodoc et al. focused on chemotherapy-induced cardiotoxicity and discussed the value of troponin as a biomarker of myocardial injury in oncologic patients [7]. The authors highlighted that troponin is a sensitive indicator of myocardial injury, as well as a marker for increased risk of developing left ventricular systolic dysfunction and progression to heart failure. The assessment of troponin levels enables early detection of cardiac injury and identification of patients that could benefit from the implementation of prevention measures. Moreover, the authors warn that troponin has no diagnostic value for cardiotoxicity and should not be used to modulate chemotherapy in cancer patients.

The cross-talk between the heart and the liver is the main focus of the articles of Cazac et al. and Gîrleanu et al. Non-alcoholic fatty liver disease reflects a disrupted lipid and carbohydrate metabolism, and it is perceived today as the hepatic manifestation of the metabolic syndrome [8]. The authors emphasize that in the presence of a common pathogenic substrate, this liver disease should be considered a marker of an increased risk for atherosclerotic cardiovascular disease. It is highlighted that patients with non-alcoholic fatty liver disease have an increased prevalence of subclinical atherosclerosis, a higher risk for the development of acute coronary syndromes (ACS), a need for more revascularization procedures, and an increased risk of fatal and non-fatal cardiovascular outcomes. 

Vascular inflammation, endothelial dysfunction, and procoagulant status are present in both liver cirrhosis and coronary artery disease [9]. Along with the main pathogenic mechanisms, the authors discuss the specifics of the diagnosis and treatment of IHD in cirrhotic patients, taking into account the fragile balance between thrombosis and bleeding, as well as the limited amount of evidence from clinical studies, especially in patients with advanced liver disease.

The link between inflammatory bowel disease and IHD is systemic inflammation. The article by Jucan et al. provides robust data regarding the elevated risk of IHD in inflammatory bowel disease patients, based on an in-depth analysis of the relevant literature [10]. The crucial role of chronic inflammation is extensively debated. The authors showed that the disruption of the intestinal mucosal barrier allows microbial translocation and other endotoxins to reach the blood. These events are followed by multi-pathway activation of systemic inflammation that leads to endothelial dysfunction, changes in the vascular smooth muscle, reduction in vessels elasticity, medial calcification, and atherosclerotic plaque formation and progression.

Cardiovascular complications of the SARS-CoV-2 infection have troubled the medical community for more than two years now, and not all questions have yet been answered. An impressive amount of data was systematized in the article by Timpau et al., covering all known mechanisms by which the infection with COVID-19 causes cardiac injury, expressed as ACS, heart failure, myocarditis, and stress cardiomyopathy [11]. The diagnostic role and prognostic value of inflammation and myocardial damage biomarkers are commented on from the perspective of an in-depth and comprehensive study of the dedicated literature, offering a robust conclusion that guides the practitioners. Duca et al. focused their research on myocardial ischemia in patients with the COVID-19 infection, and highlighted not only its complex pathophysiology, but also the diversity of its electrocardiographic expression [12]. The authors showed that myocardial ischemia is the result of endothelial damage mediated by the virus, cytokine storm caused by hyperactivation of the immune system, and oxygen supply–demand imbalance due to extensive pulmonary lesions. Moreover, they emphasized that the electrocardiographic aspects of myocardial ischemia were generally severe, and occurred even in younger and healthier patients. 

A very particular aspect regarding IHD is presented by Haliga et al., namely the ACS precipitated by carbon monoxide (CO) poisoning [13]. This original research article demonstrated that the development of myocardial injury in these patients is the combined result of hypoxia, direct CO-mediated cell damage, coronary spasm, and intracoronary thrombosis. The authors showed that ACSs occur irrespective of the poisoning severity, some in the absence of significant cardiovascular risk factors and in both normal coronary arteries and non-critical atherosclerotic plaques. Among the many interesting results of the study, we want to draw attention to the young age of STEMI patients, with a mean of 27.7 years, and the absence of comorbidities.

As medicine advances and knowledge deepens, one non-modifiable cardiovascular risk factor, namely the genetic substrate, is studied with increased interest. It seems that it will become a major landmark in the implementation of preventive measures in the future. Butnariu et al. provided a state-of-the-art analysis of the data available in the literature on the role of genetic factors in the etiology of IHD [14]. The authors discussed the phenotypic variability and the genetic heterogeneity of the disease, suggesting that genetic and genomic studies may provide the highly sought-after and awaited answers about what makes us unique in the face of the disease. The multiple facets of monogenic and polygenic coronary artery disease are emphasized, with the hope that the identification of individuals at high risk for IHD will be facilitated. The door toward the medicine of the future is now open, and gene therapy strategies no longer seem to be an unattainable desideratum.

We may conclude that this Special Issue excels through multidisciplinarity, integrating into a unified whole the experience of various specialists whose common interest is IHD. We consider that the goal has been fully achieved, and we have succeeded in drawing attention to the multiple diagnostic and therapeutic challenges determined by the association of IHD with a series of comorbidities, as well as providing useful guidance for practice. 
